# APPRECIATING TIME ALONE: AGE AND SOCIAL INTERACTION OPPORTUNITIES ENHANCE EMOTIONAL WELL-BEING WHEN ALONE

**DOI:** 10.1093/geroni/igae098.2701

**Published:** 2024-12-31

**Authors:** Anna Pot, Laura Carstensen

**Affiliations:** Stanford University, Stanford, California, United States; Stanford University, Stanford University, California, United States

## Abstract

Whereas long periods of solitude risk the health and well-being of older people, on the aggregate level, older people spend considerably more time alone than younger age groups and, on the whole, enjoy better mental health. In the present study, we consider time alone at the trait and state level. This study seeks to explore how relationships and spending time alone influences daily emotional experience in a sample spanning the adult life span. Drawing on data from an experience-sampling study that used a burst design at two timepoints five years apart, participants (N1=212 | N2=179) aged 21-94 (M1=54.58, SD1=21.95 and M2=56.80, SD2=21.24) reported their positive and negative emotions (19 in total) and whether they were alone or interacting with someone, five times daily for a week. Findings from mixed-effects models highlight a complex relationship between age, spending time alone and emotional well-being. Age positively relates to emotional well-being, and spending time alone is associated with a poorer emotional profile across the full participant sample. An interaction between age and being alone suggests that those older adults who spend relatively more time alone than average report a slight decline in emotional well-being. However, older adults who have more social interactions report significantly more positive emotions at the state level when they are alone, compared to other age groups. These results suggest that time alone may be appreciated with age, as long as social interactions do not become scarce. Findings contribute to our understanding of time spent alone and emotional well-being.

